# Predicting treatment response to ketamine in treatment-resistant depression using auditory mismatch negativity: Study protocol

**DOI:** 10.1371/journal.pone.0308413

**Published:** 2024-08-08

**Authors:** Josh Martin, Fatemeh Gholamali Nezhad, Alice Rueda, Gyu Hee Lee, Colleen E. Charlton, Milad Soltanzadeh, Karim S. Ladha, Sridhar Krishnan, Andreea O. Diaconescu, Venkat Bhat

**Affiliations:** 1 Interventional Psychiatry Program, St. Michael’s Hospital, Unity Health Toronto, Toronto, Ontario, Canada; 2 Krembil Centre for Neuroinformatics, Centre for Addiction and Mental Health, Toronto, Ontario, Canada; 3 Department of Anesthesia, St. Michael’s Hospital, Unity Health Toronto, Toronto, Ontario, Canada; 4 Department of Electrical, Computer, and Biomedical Engineering, Toronto Metropolitan University, Toronto, Ontario, Canada; 5 Department of Psychiatry, Temerty Faculty of Medicine, University of Toronto, Toronto, Ontario, Canada; 6 Institute of Medical Science, Temerty Faculty of Medicine, University of Toronto, Toronto, Ontario, Canada; PLOS: Public Library of Science, UNITED KINGDOM OF GREAT BRITAIN AND NORTHERN IRELAND

## Abstract

**Background:**

Ketamine has recently attracted considerable attention for its rapid effects on patients with major depressive disorder, including treatment-resistant depression (TRD). Despite ketamine’s promising results in treating depression, a significant number of patients do not respond to the treatment, and predicting who will benefit remains a challenge. Although its antidepressant effects are known to be linked to its action as an antagonist of the N-methyl-D-aspartate (NMDA) receptor, the precise mechanisms that determine why some patients respond and others do not are still unclear.

**Objective:**

This study aims to understand the computational mechanisms underlying changes in the auditory mismatch negativity (MMN) response following treatment with intravenous ketamine. Moreover, we aim to link the computational mechanisms to their underlying neural causes and use the parameters of the neurocomputational model to make individual treatment predictions.

**Methods:**

This is a prospective study of 30 patients with TRD who are undergoing intravenous ketamine therapy. Prior to 3 out of 4 ketamine infusions, EEG will be recorded while patients complete the auditory MMN task. Depression, suicidality, and anxiety will be assessed throughout the study and a week after the last ketamine infusion. To translate the effects of ketamine on the MMN to computational mechanisms, we will model changes in the auditory MMN using the hierarchical Gaussian filter, a hierarchical Bayesian model. Furthermore, we will employ a conductance-based neural mass model of the electrophysiological data to link these computational mechanisms to their neural causes.

**Conclusion:**

The findings of this study may improve understanding of the mechanisms underlying response and resistance to ketamine treatment in patients with TRD. The parameters obtained from fitting computational models to EEG recordings may facilitate single-patient treatment predictions, which could provide clinically useful prognostic information.

**Trial registration:**

Clinicaltrials.gov NCT05464264. Registered June 24, 2022.

## Introduction

More than 264 million people globally have depression in the context of major depressive disorder (MDD), making it the leading cause of disability worldwide [1]. Despite their common use, approximately half of patients with MDD do not respond to their first antidepressant regimen [2–4], about a third continue to be unresponsive to two or more treatment trials (i.e., treatment-resistant depression [TRD]), and it takes 4–6 weeks to discern treatment response [2]. In recent years, ketamine has attracted considerable attention for its use in MDD, including in patients with TRD. While it has been used since the 1970s as a general anesthetic [5], it has more recently been found to exert rapid antidepressant effects in people with MDD, likely via its action as a *N*-methyl-D-aspartate receptor (NMDAR) antagonist [6]. It has been shown that a subanesthetic dose of IV ketamine alleviates depressive symptoms within as little as 40 minutes of a single infusion [7], with maximum efficacy typically occurring after 24 hours [8]. Indeed, response rates of over 60% have been observed after 24 hours in patients with TRD following a single infusion of ketamine [9]. Moreover, ketamine is highly effective in reducing suicidal ideation, with this effect being largely independent from its effects on mood [10]. Although ketamine has been known to cause short-term side effects such as agitation, psychotic-like symptoms, and cognitive impairment, these side effects rarely persist more than a few hours after administration [11].

While ketamine represents a potential breakthrough treatment for TRD, numerous aspects of its therapeutic effects are yet to be understood. While ketamine quickly alleviates depressive symptoms, these effects often require repeated infusions to be sustained [9, 12]. Furthermore, some patients with TRD do not improve despite receiving multiple infusions and it is currently not possible to predict who may benefit from this intervention in advance [13]. While changes in NMDAR-related plasticity are thought to contribute to the treatment response, the underlying neural mechanisms behind response compared to relapse are not yet elucidated [14]. Of note, the antidepressant effects of ketamine may be attributed to mechanisms other than NMDAR antagonism; multiple studies have demonstrated the roles of other receptors, such as the α-amino-3-hydroxy-5-methyl-4-isoxazolepropionic acid receptor (AMPAR), in producing antidepressant effects following ketamine treatment [15].

To address the above-mentioned challenges, we aim to elucidate the neural mechanisms of ketamine treatment by modeling ketamine-induced changes in auditory evoked potentials using the auditory mismatch negativity (MMN) paradigm. The MMN is a biological index of implicit learning, occurring when a sensory stimulus violates a statistical regularity in the environment [16]; for example, when a sequence of low tones is unexpectedly interrupted by a high tone. Formally, the MMN is defined as the difference waveform obtained when subtracting the electrophysiological response to a predictable (i.e., standard) stimulus from the response to an unpredictable (i.e., deviant) stimulus. It has long served as a marker of cognitive and functional decline in psychiatric disorders, whereby a smaller MMN amplitude corresponds with poorer levels of cognitive and psychosocial functioning [17]. Furthermore, since the MMN specifically reflects the flow of current through ion channels mediated by the NMDAR and is a relatively specific measure of postsynaptic potentials produced by the binding of glutamate to the NMDAR, it could potentially serve as a biomarker for determining whether a new treatment affects NMDAR responsiveness or to predict whether a specific patient might respond favorably to such a treatment [18].

Although the MMN response can vary in patients with MDD depending on different factors such as the type of auditory deviant or the stage of MDD or co-occurring conditions, it has been demonstrated that MMN amplitudes and/or latencies are significantly impaired in patients with MDD compared to healthy controls [19, 20]. The impaired MMN reflects reduced pre-attentive cognitive processes in MDD [21–24]. Ketamine’s effects on mood have been modeled using dynamic causal modeling (DCM) [25, 26] of event-related potentials (ERPs) under the MMN paradigm [27, 28]. It has been shown that ketamine increases the auditory MMN significantly in patients with MDD [27]. DCM also revealed that this increase was related to greater modulation of forward connectivity in response to a deviant tone, which was significantly correlated with antidepressant response to ketamine [28]. In addition to feedforward connectivity, mood improvements under ketamine have been attributed to increased synaptic gain. [6]. This may reflect increased α-amino-3-hydroxy-5-methyl-4-isoxazolepropionic acid receptor (AMPAR)-related synaptic plasticity following ketamine infusions [6]. However, the neural modeling approach used thus far (i.e., DCM for ERPs) does not explicitly model the contributions of distinct receptors, including AMPARs.

This study aims to understand the antidepressant and antisuicidal effects of ketamine using predictive processing theory, a paradigm in computational and cognitive neuroscience that has also attracted attention in psychology [29]. Predictive processing theory proposes that the brain forms top-down predictions to process sensory inputs and generates bottom-up PEs to improve these predictions; this process involves primarily NMDARs and AMPARs [30]. It is hypothesized that patients with MDD fail to adapt based on PEs due to rigid predictions [30]. Thus, we propose applying a hierarchical Bayesian model, the Hierarchical Gaussian Filter (HGF) [31, 32], to model trialwise changes in MMN. Using a conductance-based neural mass model, it is possible to link the computational mechanisms of the MMN response to the underlying neural causes, allowing for the study of the effects of NMDAR antagonism on the glutamatergic system [27, 28]. Our hypothesis makes connections to Bayesian theories of brain function [25, 33], which postulate that by blocking NMDARs and stimulating AMPAR signalling, ketamine impairs top-down predictions and enhances PEs. This interaction is believed to contribute to the rapid antidepressant effects of ketamine [30]. From these computational accounts, we propose that patients with TRD exhibit deficits in top-down (higher-level) belief precision restored under ketamine via NMDAR blockade and AMPAR upregulation. Moreover, we hypothesize that precision, AMPAR- and NMDAR-related parameters will define subgroups that predict different levels of therapeutic efficacy that are maintained with repeated ketamine infusions.

### Objectives

The objective of this study is to understand the computational mechanisms underlying changes in MMN following acute and repeated ketamine intervention. Subsequently, we aim to link the computational mechanisms to their underlying neural causes and use the neurocomputational model parameters to make individual treatment predictions.

## Methods

### Participants

This will be a single-centre study of 30 participants at St. Michael’s Hospital, Unity Health Toronto, where standard of care ketamine infusions are regularly performed in a monitored setting. Patients who are scheduled to receive ketamine treatment and are interested in participating in this study will be referred to the study coordinator. Approximately six patients are scheduled to receive IV ketamine every month. Participants aged 18 to 65 years who meet the Diagnostic and Statistical Manual for Mental Disorders (DSM-5) criteria for MDD are eligible for inclusion in the study. Participants must be in a major depressive episode at the time of screening, and have a total score of ≥10 on the Montgomery-Åsberg Depression Rating Scale (MADRS) [34]. Only those who are English-speaking and competent to consent, based on their ability to provide a spontaneous narrative description of the key elements of the study, will be included. Participants must have experienced a failure of at least two trials of antidepressant therapy during the current episode (i.e., TRD) and be receiving ketamine treatment clinically as administered by their clinician. Included participants will confirm that they are on stable doses of any concomitant psychotropic medications. If any changes to concomitant psychotropic medications are required per the patient’s standard of care, the participant will be withdrawn from the study. Patients with a history of bipolar disorder, psychosis, current diagnosis of substance use disorder or history of substance use disorder during the past year, excluding nicotine and caffeine use disorder, will be excluded from participating in this study.

### Trial design

**Fig 1:**
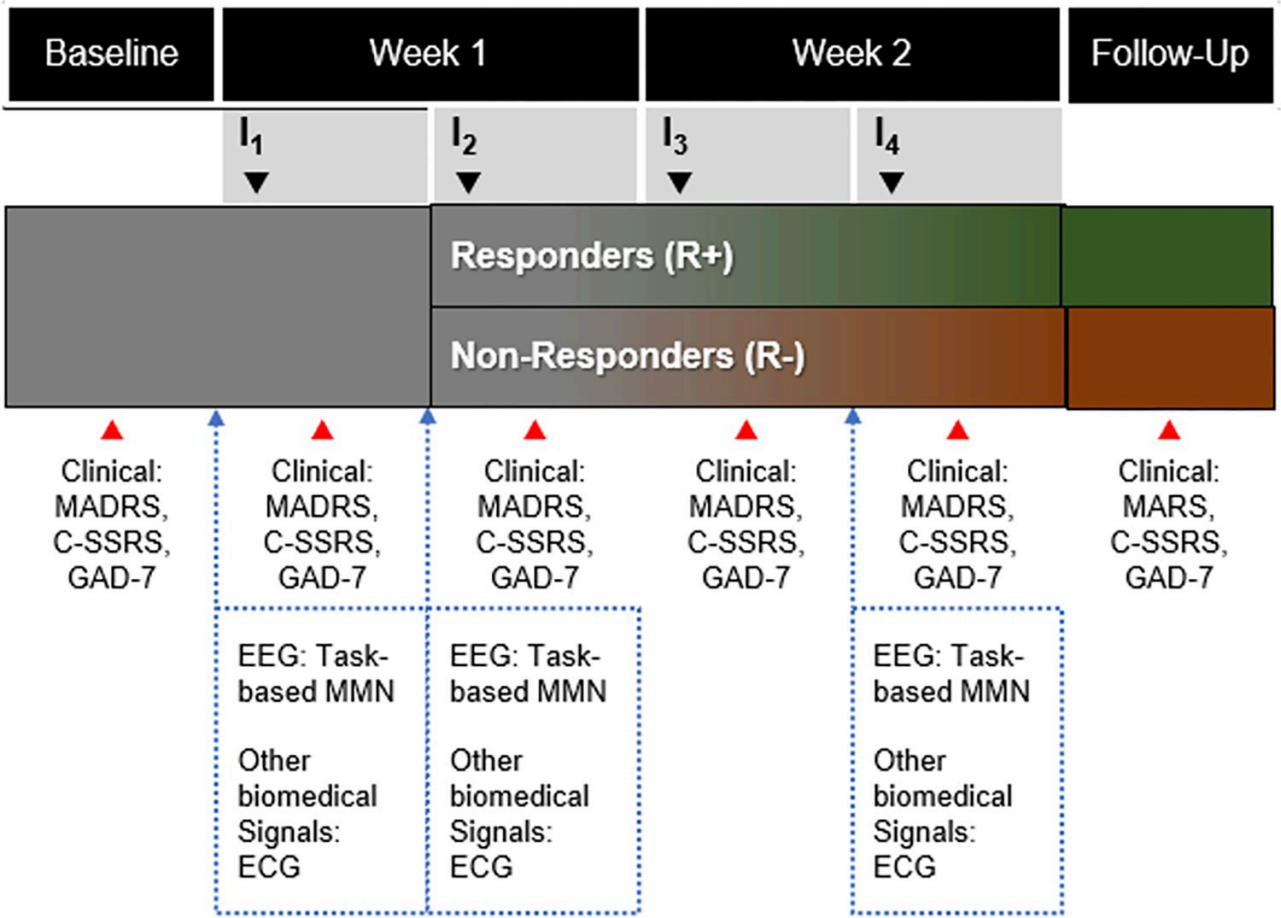
Study design.

This will be an observational study with a prospective design (Fig 1). According to the standard of care, patients will receive ketamine infusions twice a week for 2 weeks (4 total). Immediately prior to the first, second, and fourth infusions, patients will undergo an EEG scan with an auditory MMN task and simultaneous ECG recording. Clinical assessments of depression and anxiety symptoms as well as suicidal thoughts and behaviors will take place at baseline, after each ketamine infusion, and at follow-up, approximately one week after the last infusion.

### Screening and consent

Patients who are scheduled to receive ketamine treatment and have been consented to be contacted by the research team will receive a pre-screening telephone call. In a pre-screening telephone call, the study activities and timeline, as well as study goals will be explained to participants. A screening visit will be scheduled to take place over the phone. Before the screening process, the consent form will be reviewed over the phone and verbal consent will be obtained. If a participant meets the eligibility criteria for this study, they will be enrolled in the study. Participants will provide written informed consent during the first study visit.

### Sample size

We expect that at least 60% of TRD patients will respond to ketamine within the first 24 hours [35]. Furthermore, power analyses under the assumption of a medium-large effect size (i.e., Cohen’s *f* = 0.4) based on previous studies [32, 36, 37] and a 15% dropout rate indicate that N = 30 participants in total will be sufficient to test our hypotheses.

### Retention strategy

Participants will be compensated CAD $20 per in-person EEG visit, for a total of CAD $60. The research staff will give each participant a reminder call before each recording session. Participants will no longer be eligible to continue the study if they miss more than one infusion.

### Data collection

#### Clinical outcomes

This study will evaluate depressive symptoms, anxiety symptoms, and suicidal risk at baseline, after each ketamine infusion, and at the one-week follow-up visit. These assessments will be conducted using the MADRS, Columbia Suicide Severity Rating Scale (C-SSRS), and Generalized Anxiety Disorder 7-item (GAD-7). The MADRS [34] is a 10-item clinician-rated scale designed to measure MDD severity and detect changes due to treatment. Suicide risk will be examined using MADRS item 10 and the C-SSRS [38], a semi-structured clinical interview, which utilizes a set of prompts and questions to obtain more complete information on events suggestive of suicidality. Additionally, the GAD-7 [39] is a 7-item self-rated questionnaire designed for assessing generalized anxiety disorder.

#### EEG and ECG

EEG and ECG data will be collected immediately before the first, second, and fourth treatment visits. A Biosemi ActiveTwo system will be used to collect EEG data at a sampling rate of 512 Hz during the auditory MMN task. A 32-electrode configuration based on the extended 10–20 system, an internationally recognized method for electrode placement, will be utilized. To accurately identify and correct eye movement artifacts, the horizontal and vertical electro-oculogram (EOG) measures will be recorded through electrodes placed lateral to each eye, as well as electrodes placed below each eye. Reference electrodes will be placed bilaterally on the mastoids. The scalp electrodes will be affixed using a cap, and a water-based conductive gel will be applied to ensure optimal conductivity and signal clarity. The ECG signal will be recorded with an additional electrode, part of the EEG system, attached on the participant’s wrist. This setup is designed to obtain a high-quality data acquisition without potential physiological interference.

#### MMN task

The auditory MMN paradigm has been previously used in MDD patients following ketamine interventions [40, 41], and was shown to be safe and have no impact on symptoms. EEG data will be collected during an MMN paradigm specifically designed to minimize the correlation between low-level and high-level precision-weighted prediction errors (for details, see Weber et al. [42]).

**Fig 2:**
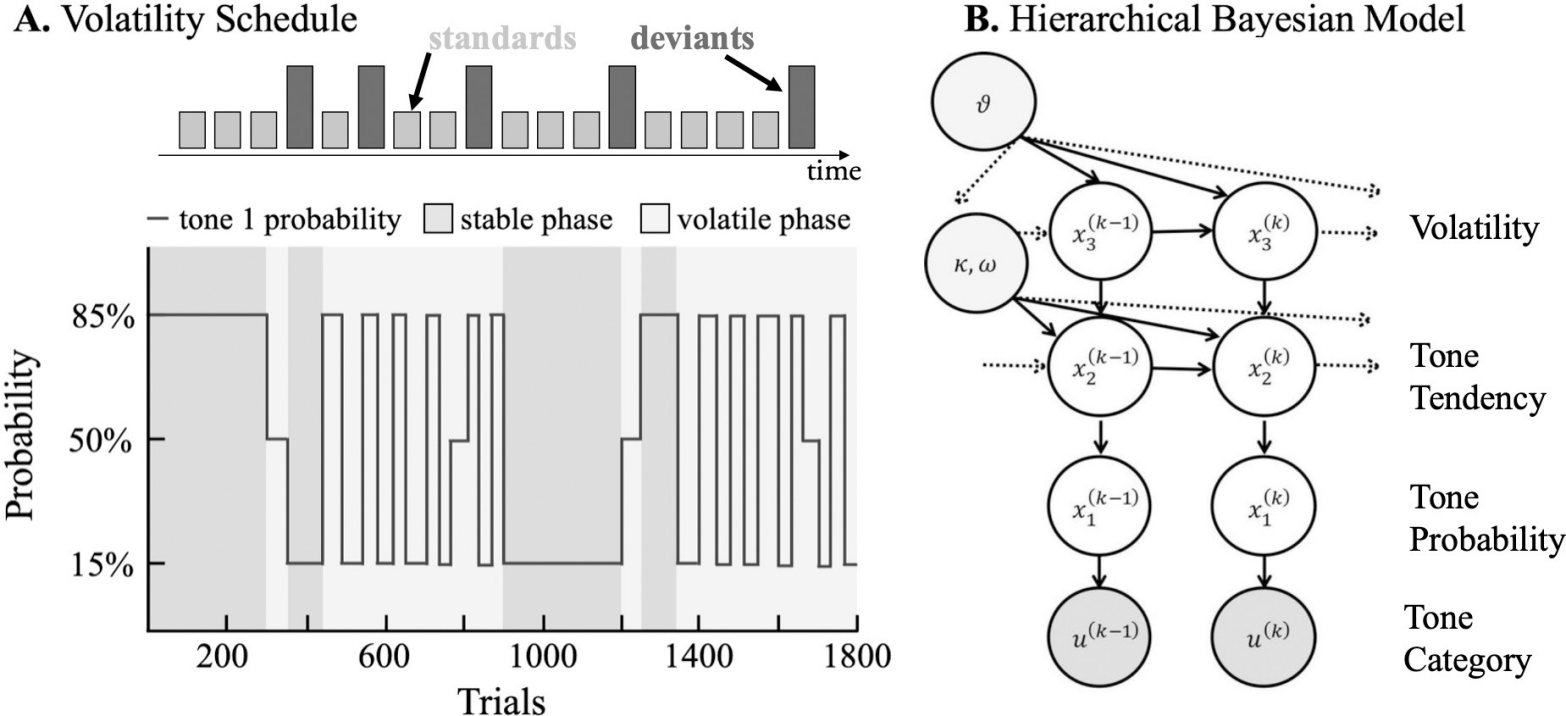
Auditory MMN task. (A) The auditory MMN task consists of a train of alternating tones of different frequencies, and the probability of the tones is systematically varied with a given probabilistic schedule including alternating stable and volatile phases. (B) Three-level hierarchical Gaussian filter (HGF) binary perceptual model, a Hierarchical Bayesian Model, is used to model the effect of ketamine on MMN.

This auditory MMN task consists of a train of alternating tones of different frequencies (528 Hz versus 440 Hz) presented for 70 ms including 5 ms fade-in and 5 ms fade-out (Fig 2). To investigate individual differences in the expression of hierarchical precision-weighted prediction errors (PEs), we will systematically vary the probability of the tones. The task consists of two types of phases: stable phases were defined as periods where the probability of hearing the same tone remained constant for at least 90 trials and volatile phases were all other phases (see volatility structure in Fig 2A). Auditory stimuli will be presented using PsychToolbox (PTB3, psychotoolbox.org) and participants will be directed to look at a fixation point for the duration of the experiment, which lasts a total of 20 minutes.

### Data analysis

#### Clinical outcomes

Response will be defined as a reduction of 50% or more in the total scores of both the MADRS and GAD-7, and a 50% or greater reduction in item 10 of the MADRS scale, from baseline to the follow-up. Remission will be defined as a MADRS total score of 10 or below, assessed at the follow-up visit. C-SSRS and GAD-7 scores will be utilized to examine the association between biomedical signals and suicide and anxiety, respectively.

#### EEG quality control and assurance procedures

Pre-processing and data quality control will be performed using established pipelines based on the Statistical Parametric Mapping toolbox (SPM12; RRID:SCR_007037) [43]. In brief, continuous EEG recordings will be referenced to the average, high-pass filtered using a Butterworth filter with cutoff frequency 0.5 Hz, down-sampled to 256 Hz, and low-pass filtered using Butterworth filter with cutoff frequency 35 Hz. The data will be epoched into 500 ms segments starting at a pre-stimulus baseline of 100 ms.

We will reject all trials overlapping with eye blink events, as detected by a thresholding routine on the vertical EOG channels, as previously used to model the effects of ketamine on sensory learning in a placebo-controlled study with healthy volunteers [44]. Finally, an artifact rejection procedure will be applied using a thresholding approach on all EEG channels to detect problematic trials or channels. Trials in which the signal recorded exceeds 75 μV relative to the pre-stimulus baseline will be removed from subsequent analysis, and channels in which more than 20% of trials need to be rejected will be marked as bad and subsequently interpolated for sensor-level statistics. Continuous ECG data will be analyzed using the same SPM12-based pipelines [43]. The ECG signal will provide information about the patient’s emotional state besides helping with removing cardiac interference on the EEG.

#### Neurocomputational mechanisms and model

**Fig 3:**
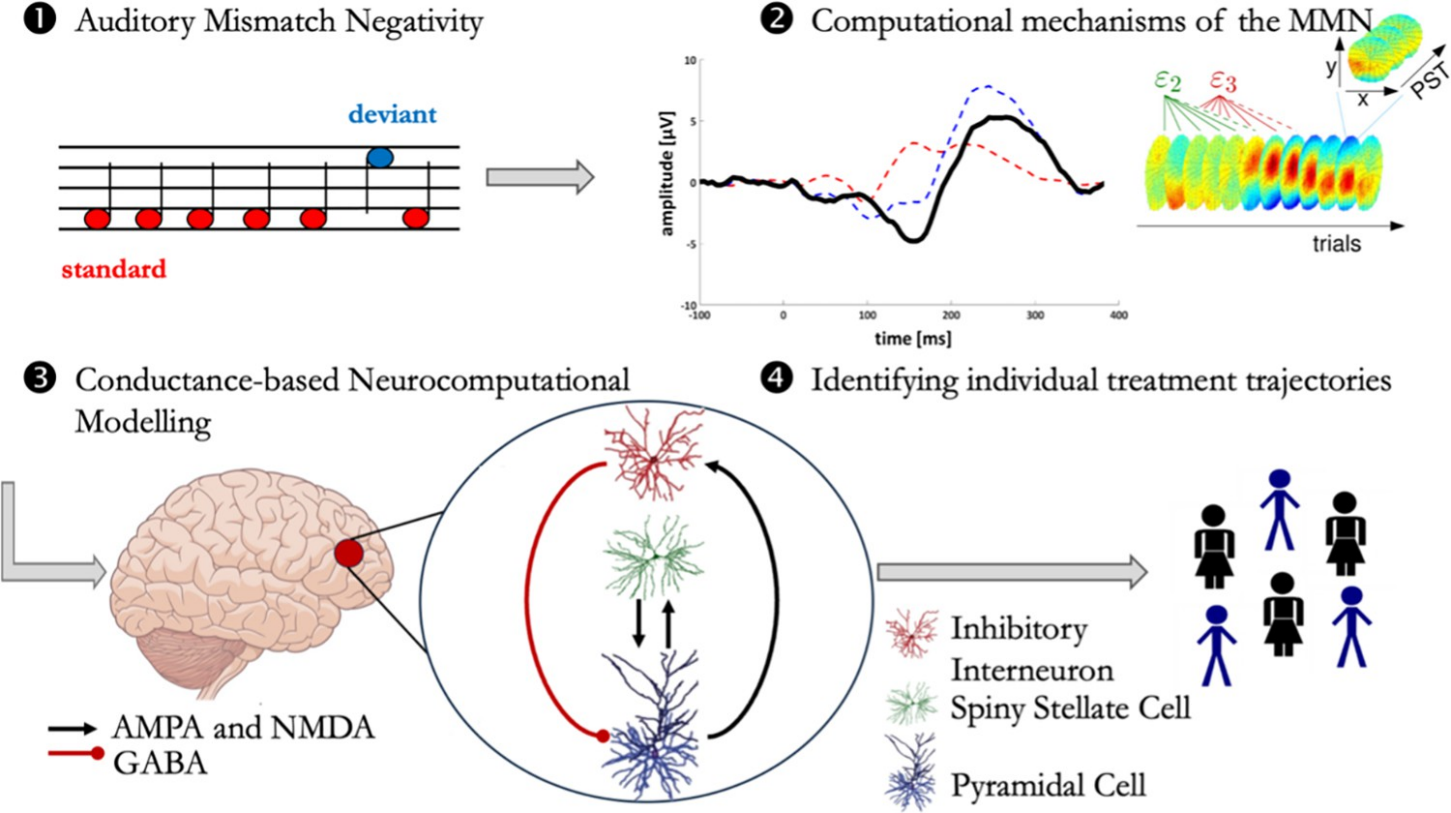
Predicting antidepressant ketamine effects in TRD using a conductance-based neural mass model. This flowchart provides a step-by-step approach to modeling ketamine effects of perceptual inference using the auditory MMN paradigm and EEG recordings. 1) The auditory MMN, which occurs when a sequence of low tones (predictable stimulus or standard) is unexpectedly interrupted by a high tone (unpredictable stimulus or deviant). 2) The second step includes specifying the generative model, which embodies a probabilistic forward mapping from hidden brain states to observed auditory MMN. 3) The model can be inverted and applied to data for inferring underlying individual pathophysiology. (3-population neural model, with inhibitory interneuron, spiny stellate cell, and pyramidal cell populations in layer IV cortical column drawn using Adobe Illustrator). For illustration purposes, intrinsic glutamatergic connections mediated by the AMPA and NMDA receptors are grouped. 4) The utility of these models for detecting physiologically defined subgroups will be tested in TRD patients.

Fig 3 depicts the process of analyzing the EEG signals from the MMN task. To translate the effects of ketamine on the MMN to computational mechanisms, we will model changes in the auditory MMN using the HGF [31], a Bayesian model which assumes that individuals learn not only about the probability of a stimulus, but also about the volatility of the environmental context (Fig 2B). Parameters capturing hierarchical learning during the auditory MMN task will be extracted after fitting the model to each individual’s data. These parameters will be obtained at infusion Visits 1, 2, and 4.

To link the identified computational mechanisms to their neural causes, we will apply a conductance-based neural mass model to model the cortical generators of the MMN signal [45]. Specifically, this biophysically interpretable model will allow us to investigate altered densities of specific receptor types, including NMDAR, AMPAR, and gamma-aminobutyric acid receptor (GABAR). This model assumes that cortical generators of the EEG signal can be modeled as simplified cortical columns with three neuronal populations comprising pyramidal cells, inhibitory interneurons, and spiny stellate cells. This source model rests on Morris-Lecar equations, which are extensions of the Hodgkin and Huxley’s model, scaled up for EEG analysis using the Fokker-Planck formalism [45], and parametrizes conductance properties of three different receptor types: NMDAR, AMPAR, and GABAR [30].

Electrophysiological data will be analyzed using computational models assessing disruptions in effective connectivity and NMDAR plasticity during perceptual inference [25, 33] using SPM12. This computational framework capitalizes on previous work, which has established close links between NMDAR plasticity and hierarchical precision-weighted PE learning [44, 46]. We will investigate effective connectivity and altered densities of specific receptor types (e.g. NMDAR and AMPAR) that have been implicated in pharmacological studies using MMN [47, 48].

### Data management and quality control

Data will be entered into an encrypted and password-protected Microsoft® Excel database (Microsoft Corp., Redmond, WA, USA) by qualified research personnel. Appropriate range and missing data filters will be used to address data quality. Data accuracy will be assessed by random selection of 10% of individuals who will have their data confirmed by a second reviewer. Following the acquisition of the EEG data, the research staff will ensure that data is de-identified. Continuous EEG data will be de-identified by using only the participant study IDs and the date and time of measurement. All EEG data headers will be stripped of potentially identifying information to further protect participants. The confidentiality of the data collected and the identity of the individuals participating in this study will be strictly maintained. All files pertaining to subjects in the study will be coded numerically.

### Ethics statement

This study was approved by the Unity Health Toronto Research Ethics Board (protocol number 22–084). Written informed consent will be obtained from participants prior to data collection. Recruitment began on December 6, 2022 and is currently ongoing.

## Discussion

While ketamine has demonstrated considerable efficacy in the treatment of depression, some patients do not respond and it is not currently possible to predict who will benefit from this treatment [49]. Moreover, the exact mechanisms by which ketamine exerts antidepressant effects are not fully understood [50]. Based on predictive coding theories of neurocomputation [25, 33, 51], we hypothesize that ketamine’s antidepressant effects are driven by a reduction in precision due to NMDAR-antagonism and that, in turn, this potentiates learning rates and long-term plasticity expressed as increases in the conductance and effective connectivity parameters of our model.

Beyond uncovering the mechanisms of ketamine’s action, the computational parameters derived from fitting our models to patient behavior and EEG recordings may hold predictive value for individual treatment outcomes. If validated, this approach could offer valuable prognostic insights, helping to tailor treatment plans based on specific neurophysiological markers. Our modeling framework acknowledges the complex and multifaceted nature of TRD, encompassing its physiological, psychological, and social dimensions. By integrating these factors within our analysis approach, we can explore how they interact and contribute to the overall treatment response.

One limitation of this study is the lack of follow-up EEG recordings. As the last EEG recording takes place immediately before the final ketamine infusion, we will not be able to assess the effects of the final ketamine infusion on EEG measures. Additionally, we will not be able to include the long-term effects of ketamine treatment on biomedical signals in our model as we will not collect those signals at follow-up. Future studies should consider repeated follow-ups post-treatment, as this would allow for an understanding of the long-term effects of ketamine in responders and non-responders as well as the changes that underlie remission following an extended period without ketamine infusions.
